# *Pantoea* sp. Isolated from Tropical Fresh Water Exhibiting *N*-Acyl Homoserine Lactone Production

**DOI:** 10.1155/2014/828971

**Published:** 2014-08-12

**Authors:** Wen-Si Tan, Nina Yusrina Muhamad Yunos, Pui-Wan Tan, Nur Izzati Mohamad, Tan-Guan-Sheng Adrian, Wai-Fong Yin, Kok-Gan Chan

**Affiliations:** Division of Genetics and Molecular Biology, Institute of Biological Sciences, Faculty of Science, University of Malaya, 50603 Kuala Lumpur, Malaysia

## Abstract

*N*-Acyl homoserine lactone (AHL) serves as signaling molecule for quorum sensing (QS) in Gram-negative bacteria to regulate various physiological activities including pathogenicity. With the aim of isolating freshwater-borne bacteria that can cause outbreak of disease in plants and portrayed QS properties, environmental water sampling was conducted. Here we report the preliminary screening of AHL production using *Chromobacterium violaceum* CV026 and *Escherichia coli* [pSB401] as AHL biosensors. The 16S rDNA gene sequence of isolate M009 showed the highest sequence similarity to *Pantoea stewartii* S9-116, which is a plant pathogen. The isolated *Pantoea* sp. was confirmed to produce *N*-3-oxohexanoyl-L-HSL (3-oxo-C6-HSL) through analysis of high resolution mass tandem mass spectrometry.

## 1. Introduction

Quorum sensing (QS) expresses the mechanism in which gene expression is regulated in relation to cell-density dependent manner to induce concerted physiological process in bacterial community [1, 2]. The concerted action by bacterial communication in relation to population density is coupled with the production of signaling molecules which function as “autoinducers” [1]. There are two classified autoinducers, namely, autoinducer-1 and autoinducer-2 [1, 3]. Gram-negative bacteria utilize the autoinducer-1 involving* N*-acyl homoserine lactone (AHL) and Gram-positive bacteria utilize the posttranslationally modified oligopeptide signaling molecules classified as autoinducer-2 [1, 4]. To date, the only shared QS mechanism for both Gram-positive and Gram-negative bacteria involves the autoinducer-2 production by the enzyme LuxS [5, 6].

Autoinduction of bacterial bioluminescence was first discovered in the early 1970s when this discovery became the focus of the QS mechanism [7, 8]. The marine symbiotic* Vibrio fischeri* and its free living relative* V*.* harveyi* are able to induce bioluminescence in a cell density-dependent manner. The AHL molecules which have been studied intensely are utilized by a vast majority of Gram-negative bacteria typically dependent on a “LuxI” autoinducer synthase and a cognate “LuxR” transcriptional activator protein [9]. When AHL threshold level is achieved, the signaling molecules will bind to the transcriptional activator (LuxR protein), which will then form a AHL/LuxR complex and regulate expression of target genes [10, 11].

Since QS play an important role in bacteria cell-to-cell communication, it is therefore important for us to study the fresh water inhabiting bacteria which exhibits QS properties [12, 13]. Therefore, it raises significant interest on the presence of QS phytopathogen in aquatic environment as river water is commonly used for irrigation. Presence of phytopathogen may have implication in the agriculture if the QS phytopathogens-contaminated fresh water is used for crop irrigation. Waterfall is chosen as sampling source for bacteria isolation. There are many waterfalls that are located inside tropical rainforest in Malaysia and Sungai Tua waterfall is the interest in our study as it is a touristic site for locals. In view of this, we investigated the presence of QS bacteria in Malaysia tropical rainforest waterfall sample and we report the isolation of plant pathogen that portrayed QS properties, namely,* Pantoea* sp. Plant pathogen that causes diseases to major crops could be water-borne [14].

## 2. Experimental Section 

### 2.1. Sample Collection and Bacterial Isolation

Water sample was collected from the top of Sungai Tua waterfall during October 2013. The waterfall was located 10 km from Selayang and Ulu Yam with GPS coordinates of N03 19.91′ E101 42.15′. The sample was collected below water surface at the depth of 12 cm. Sample was collected in sterilized plastic tubes and kept at 4°C till further processing [15]. The water sample was then serially diluted with sterile saline and spread onto Reasoner's 2A agar (0.5 g/L proteose; 0.5 g/L casamino acids; 0.5 g/L yeast extract; 0.5 g/L dextrose; 0.5 g/L soluble starch; 0.3 g/L dipotassium phosphate; 0.05 g/L magnesium sulfate; and 0.3 g/L sodium pyruvate) and incubated overnight (24 h) at 28°C. The observable different morphologies of the bacteria colonies were isolated. Pure colony was obtained with several passages on Trypticase Soy (TS) medium (10 g/L tryptone; 5 g/L soytone; 5 g/L NaCl; and 15 g/L Bacto agar).

### 2.2. Bacterial Strains, Culture Conditions, AHL Biosensor Assay, and Controls for Short Chain AHLs

Bacterial isolate M009 isolated from waterfall sample was selected for further Analysis and cultured on TS medium. Two different AHL biosensors (*Chromobacterium violaceum* CV026 and* Escherichia coli* [pSB401]) were used for the preliminary screening of QS signaling molecules. Both the biosensors respond to the presence of short chain AHL molecules in which CV026 produced purple violacein pigmentation while* E. coli* [pSB401] respond by induction of luminescence [16, 17]. In the AHL preliminary screening,* Erwinia carotovora* GS101 and* E. carotovora* PNP22 were used as positive and negative controls, respectively [18]. Lysogeny broth (LB) medium (10 g/L tryptone; 5 g/L yeast extract; 5 g/L NaCl; and 15 g/L Bacto agar) was used as growth medium for routine culture of* C. violaceum* CV026,* E. coli* [pSB401],* E. carotovora* GS101, and* E. carotovora* PNP22.

### 2.3. Preliminary Screening of AHLs Using Bacterial Biosensors

Isolate M009 was screened for AHL production by cross streaking the bacterial isolates with CV026 on a LB agar plate (24 h at 28°C). Secondly,* E. coli* [pSB401] was used as an AHL biosensor to screen for the production of AHL. After 24 hours incubation at 28°C, the photon camera with 60 s of exposure was used to observe the bioluminescence induced [19].

### 2.4. Strains Identification

Molecular identification of isolate M009 was done by analyzing the PCR-amplified bacterial 16S rDNA gene. The primer pair 27F-1525R [20, 21] was used for PCR amplification using PCR mix from Promega (Promega Kit, Madison, WI, USA). Prior to that, genomic DNA of isolate M009 was extracted using MasterPure^TM^ DNA Purification Kit (Epicentre Inc., Madison, WI, USA). PCR amplification and amplicons purification were conducted as described previously and the PCR product sequence alignment was done using GenBank BLASTN program followed by phylogenetic tree reconstruction using the Molecular Evolutionary Genetic Analysis version 6.0 [22, 23].

### 2.5. AHL Extraction

Isolate M009 was cultured in LB broth buffered to pH 5.5 with 50 mM of 3-(*N*-morpholino) propanesulfonic acid (MOPS) in an incubator shaker (200 rpm; 28°C; 18 h) [24]. The cultured supernatant was extracted twice with equal volume of acidified (0.1% v/v glacial acetic acid) ethyl acetate as described previously [24]. The organic solvent was dried in fume hood and the dried extracts were resuspended in 1 mL of acidified ethyl acetate and desiccated completely. Finally, 200 *μ*L of acetonitrile (HPLC grade) was added and vortexed to dissolve the dried extracts. The extract was then centrifuged at 12,000 rpm for 5 min to remove any insoluble residue. The dissolved sample with 75 *μ*L aliquot was withdrawn from the upper layer and inserted in sample vials for mass spectrometry Analysis.

### 2.6. AHL Profiling by Mass Spectrometry (MS)

AHL profile was analyzed by MS as described previously [24]. The Agilent RRLC 1200 system was utilized as the liquid chromatography (LC) delivery system with the use of Agilent ZORBAX Rapid Resolution HT column (2.1 mm × 100 mm, 1.8 *μ*m particle size) for separation of AHL molecules. The flow rate for the Analysis was set to 0.3 mL/min at 60°C and injection volume was adjusted to 20 *μ*L. The mobile phases A (LCMS grade water with 0.1% v/v formic acid) and B (HPLC grade acetonitrile with 0.1% v/v formic acid) were prepared and set a ratio of 80 : 20, respectively. The high resolution electrospray ionization mass spectrometry (ESI-MS) was performed with the Agilent 6490 Triple Quadrupole LC/MS system and was carried out in the ESI-positive mode (probe capillary voltage 3 kV, nebulizer pressure at 20 psi, sheath gas at 11 mL/h, desolvation temperature 200°C, collision energy, and fragmentation at both 5 eV and 380 eV, resp.). The precursor ion scan mode targeting at the production ion with* m/z* 102 indicates that [M+H]^+^ ion of the core lactone ring moiety. The* m/z* value range to detect the precursor ions was set at* m/z *150–400. To analyse the MS spectra, we used Agilent MassHunter software.

## 3. Results and Discussion

### 3.1. Strains Isolation and Identification

This study aimed to isolate AHL producing bacterial isolate from the tropical rainforest waterfall. Irrigation systems carry water directly from river to farms whereby the usual source is from waterfall [25]. The temperature of the water was 25°C at daytime and the pH gave a reading value of 7 at the point of the sampling. The water sample was collected at the most top of the waterfall where human activities were less common in order to reduce the faecal contamination during water collection [25, 26].

**Figure 1:**
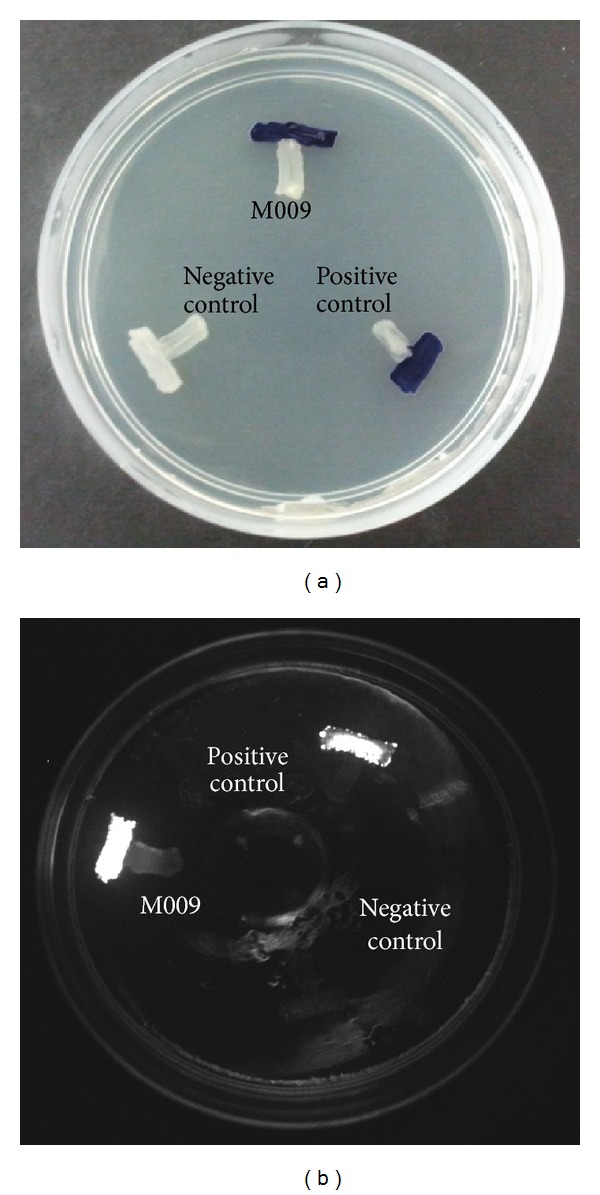
(a) AHL screening of strain M009 with CV026.* E. carotovora* PNP22 (Negative control) devoid of QS activity was included and* E. carotovora* GS101 (Positive control) that can activate CV026 was included for comparison. Both positive control and strain M009 induce the purple violacein pigmentation. (b) AHL screening of strain M009 with* E. coli* [pSB401].* E. carotovora* PNP22 (negative control) and* E. carotovora* GS101 (positive control) served as negative and positive controls, respectively. The positive control and strain M009 exhibit bioluminescence.

The number of QS bacteria identified has been increased due to the availability of AHL biosensors which are able to detect the presence of AHLs [6, 27]. The AHL biosensors practically rely on LuxR proteins and display specificity towards the cognate AHL and positively regulate the transcription of a reporter gene [27]. The biosensor* C. violaceum* CV026 responds to AHL with* N*-acyl chain length of C4 to C8 that will induce purple violacein pigmentation [16]. It is a commonly used bacterial biosensor due to rapidness and accuracy of AHL detection. On the other hand,* E. coli* [pSB401] biosensor will exhibit luminescence due to the presence of short chain AHLs and it is most sensitive to cognate 3-oxo-C6-HSL [17]. In this study, both CV026 and* E. coli* [pSB401] were employed for preliminary screening of AHLs produced by strain M009 (Figure 1).

Preliminary screening of the AHL production with CV026 (Figure 1(a)) and* E. coli* [pSB401] (Figure 1(b)) indicated that isolate M009 strain produced short chain AHL [16, 17]. This strain was then sent for molecular identification.

### 3.2. Molecular Bacterial Identification

**Figure 2:**
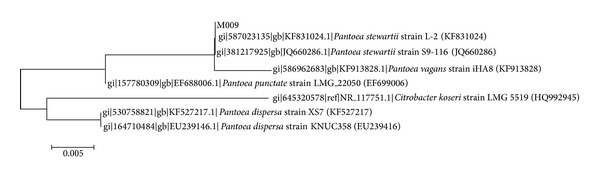
Phylogenetic tree reconstructed using maximum likelihood algorithm showing phylogenetic relationships of isolate M009 to members of other species of the genus* Pantoea*. There are a total of 1220 positions in the final dataset. Bar, 5 substitutions per 1000 nucleotide positions.

Isolate M009 identity was confirmed by analysis of its 16S rDNA gene nucleotides sequences showing that it clusters closely with the* Pantoea* genus showing 98-99% similarity in the BLAST search. The nucleotide sequences were subsequently deposited into GenBank with the accession number KJ830125. According to the phylogenetic tree constructed (Figure 2), with the evolutionary history which was inferred by using the Maximum Likelihood algorithm based on the Tamura-Nei model [1], strain M009 is identified as* Pantoea stewartii*, a plant pathogen. The tree with the highest log likelihood (−2388.7284) is shown. Initial tree(s) for the heuristic search were obtained automatically as follows. When the number of common sites was < 100 or less than one fourth of the total number of sites, the maximum parsimony method was used; otherwise BIONJ method with MCL distance matrix was used. There were a total of 1,221 positions in the final dataset. Evolutionary analyses were conducted in MEGA6.

### 3.3. Assay of AHL from Supernatant

*Pantoea stewartii* is causative agent of Stewart's wilt, a bacterial disease transmitted by the corn flea beetle mainly sweet corn (*Zea mays*). It has been classified as a quarantine microorganism and must be differentiated from other yellow enteric bacteria that occur frequently with corn [28]. Stewart's wilt is also a problem on certain elite inbred maize lines used for producing hybrid field corn seed and more than 60 countries place import regulations on maize seed imports from affected areas to prevent the distribution of pathogen and to reduce the influence towards agriculture economy [28]. Thus the availability to detect AHL profile of* Pantoea* sp. could be a milestone in understanding this potential plant pathogen.

**Figure 3:**
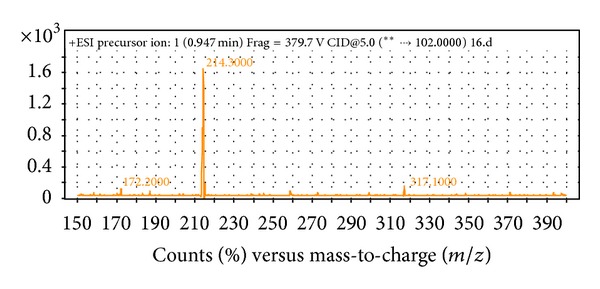
Mass spectrometry analysis of AHL extracts from* Pantoea* sp. strain M009. Mass spectra of 3-oxo-C6 HSL (*m/z* 214.3000) was detected.

The spent culture supernatants of M009 strain were analyzed using the Agilent 6500 Q-TOF LC/MS system and mass spectrometry analysis. The presence of 3-oxo-C6-HSL is confirmed (Figure 3) and this is the type of AHL which is identical to the* V. fischeri *autoinducer [11–13]. Our result is also in agreement with other report work that showed* P. stewartii *primarily produces 3-oxo-C6-HSL molecules [29, 30]. The AHL synthase and cognate AHL receptor for 3-oxo-C6-HSL in the reported* P. stewartii *are* EsaI *and* EsaR* which are the homologues of* luxI *and* luxR *[30]. The similarity of these results further confirmed that bacterial communication occurs on plant surfaces and participants likely include the signal producers community, signal eavesdroppers, and plant host [4]. In addition, our group is currently expanding this work to the whole genome sequence research to gain more insights into this strain particularly to isolate the* luxI *and* luxR* homologues of our isolate.

QS regulates a battery of bacterial pathogenicity factors [30] and, hence, this work facilitates the evidence to illustrate the importance to emphasize the research on AHL-producing plant bacteria that are present in the environment.* EsaI* which produces 3-oxo-C6-HSL in P. stewartii has been shown to regulate extracellular polysaccharide capsule production and pathogenicity [30]. This work suggests that fresh water may be a potential reservoir for QS pathogens that should be given attention by more intense research.

## 4. Conclusion

We reported here the production of 3-oxo-C6-HSL in* Pantoea* sp. M009 strain isolated from fresh water sample. This discovery could form the basis for understanding the communication system of this isolate and relate it to QS-modulated virulent determinants.
